# Association analysis of whole genome sequencing data accounting for longitudinal and family designs

**DOI:** 10.1186/1753-6561-8-S1-S89

**Published:** 2014-06-17

**Authors:** Yijuan Hu, Qin Hui, Yan V Sun

**Affiliations:** 1 Department of Biostatistics and Bioinformatics, Emory University, Atlanta, GA, USA; 2 Department of Epidemiology, Emory University, Atlanta, GA, USA; 3 Department of Biomedical Informatics, Emory University, Atlanta, GA, USA; 4 Center for Health Research, Kaiser Permanente Georgia, Atlanta, GA, USA

## Abstract

Using the whole genome sequencing data and the simulated longitudinal phenotypes for 849 pedigree-based individuals from Genetic Analysis Workshop 18, we investigated various approaches to detecting the association of rare and common variants with blood pressure traits. We compared three strategies for longitudinal data: (a) using the baseline measurement only, (b) using the average from multiple visits, and (c) using all individual measurements. We also compared the power of using all of the pedigree-based data and the unrelated subset. The analyses were performed without knowledge of the underlying simulating model.

## Background

Whole genome sequencing (WGS) makes it possible for investigators to extend association studies to rare variants. Rare variants, which have minor allele frequencies (MAFs) of less than 1% to 5%, might play an important role in the etiology of complex traits and account for missing heritability unexplained by common variants [1-3]. However, traditional single-variant tests for common variants have limited power for testing rare variants because of their low frequencies and large numbers. A number of methods [4-7] have been developed to address this challenge by jointly analyzing rare variants within a region. Among these methods, the burden test of Lin and Tang [7] easily fits into the regression framework that can accommodate complex study designs and phenotypes, thus remaining a competitive option.

The longitudinal study design, which collects repeated measurements on the same subject over time, has been routinely used in epidemiologic and clinical research. The repeated measurements can reduce error and thus increase statistical power compared with the single measurement. There have been increasing applications of such a design in genome-wide association studies (GWAS) with a focus on common variants. The analytical strategies include using the measurement at a single time point [8], using the summarized univariate measurement [9] and adopting the linear mixed-effects model to fully exploit information in the repeated measurements [10,11]. However, the implementation of such designs is limited in the context of WGS studies with a focus on rare-variant associations.

In the era of next-generation sequencing studies, the family-based design has the unique advantages of protecting against population stratification, detecting genotyping errors, and facilitating accurate imputation, all of which are challenging issues to cope with in the studies of rare variants using unrelated subjects. However, it is well known that enrolling and sequencing additional family members will not increase statistical power as much as that can be achieved by the same number of unrelated subjects. The degree of power gain from the added family members depends on the extent of the within-family correlation and the size of each family. Thus, it is of interest to assess this power gain in each specific study.

Genetic Analysis Workshop 18 (GAW18) provided WGS data (sequencing plus imputation) in a pedigree-based sample with longitudinal measurements for systolic blood pressure (SBP) and diastolic blood pressure (DBP). In this study, we implement methods to exploit longitudinal and family structures and apply these methods to examine the associations of aggregated rare variants, as well as common single-nucleotide polymorphisms (SNPs), with SBP and DBP. We compare the power of the methods using the baseline measurement only, using the averaged value over repeated measurements, and using the full information of longitudinal data. We also contrast the power of the methods using all of the pedigree-based data with that using the unrelated subset.

## Methods

In this study, we focus on the first replicate of simulated SBP and DBP and the genetic data from sequencing and imputation only on chromosome 3 because of limited computation resources. The analyses were performed without knowledge of the underlying simulating model. We obtained 849 individuals from 20 pedigrees, among which 142 are unrelated. All individuals have SBP and DBP measurements at three time points with no missing data, as well as age, gender, smoking status, and antihypertensive medication status. Note that we preadjust the SBP and DBP measurements by the antihypertensive medication status (i.e., increasing SBP by 10 mm Hg and DBP by 5 mm Hg if the subject is taking medication). We define common variants as those with MAFs 5% or greater and obtain 403,098 SNPs on chromosome 3. We jointly analyze rare variants by mRNA transcripts, which are the functional products of genes. We exclude transcripts whose total rare allele frequency (i.e., sum of MAFs over all inclusive variants) is less than 0.01 and end up with a total of 813 transcripts represented by accession numbers. Given a common single-nucleotide polymorphism (SNP) or a transcript for the phenotype, we consider using (a) the baseline, (b) the time-averaged, and (c) the repeated measurements; for study subjects, we consider using (a) the entire pedigree-based sample and (b) the unrelated subjects only. All statistical analyses were carried out in R (http://www.r-project.org) version 2.15.1.

### Notation

Assume there are *m *rare variants in a transcript. Under the population-based design, let *Y_it _*denote the phenotype measured for subject *i *at time *t, **G***_*i *_= (*G*_*i1*_ , *G*_*i2*_ , ..., *G*_*im*_ )^T ^the genotypes of the *m *variants, and ***X***_*it *_= (*X*_*it1*_ , *X*_*it2*_ , ..., *X*_*itq*_ )^T ^the *q *covariates including the intercept and possibly time-varying ones. Under the family-based design and when subject *i *belongs to family *p*, we modify the aforementioned notation to be *Y_pit_*, ***G***_pi_ and ***X***_*pit*_ .

### Burden score of rare variants

We focus on variants with MAF less than 5% and that are putatively functional (i.e., nonsense, missense, or splice site mutations). Specifically, using the chromosomal location (NCBI built 37.1) provided by the GAW18 data, we search for functional annotation of 812,234 SNPs with MAFs 5% or less on chromosome 3 using the GVS server (http://snp.gs.washington.edu). We consider the following functional categories: missense, missense-near-splice, splice-3, splice-5, stop-gained, stop-lost, and stop-lost-near-splice. Note that the functional annotation is specific to each transcript. The burden score of the *i*th subject at a given transcript is defined as the sum of genotypes of the selected variants: $S_{i}={\sum {}_{j=1}}_{,\ldots ,m}G_{ij}$.

In the framework of Lin and Tang [7], the burden score is used in the following regression models as a regular covariate.

### Models and assumptions

#### Unrelated subjects with single measurements of blood pressure

The single measurements of blood pressure can be the baseline or time-averaged SBP or DBP. We denote the baseline and averaged measurements by *Y_i1 _*and $Y_{i+}=\sum_{t}Y_{\mathrm{it}},$ respectively. Because they are all quantitative, it is natural to relate each of them to *S_i _*and ***X***_*i *_through the linear regression model:

(1) $$Y_{i1}\;\text{or}\;Y_{i+}=bS_{i}+g^{\text{T}}X_{i}+e_{i},$$

where $\epsilon_{\text{i}}$ is an error term with mean zero and variance $\sigma^{\text{2}}$ and ***X***_*i *_includes the intercept, gender, smoking status, and baseline (averaged) age in the baseline (averaged) phenotype model.

#### Unrelated subjects with repeated measurements of blood pressure

To account for the correlation of measurements from the same individual, we use the linear mixed-effects model. For subject *i *at time *t*, it is written as

(2) $$Y_{it}=bS_{i}+g^{\text{T}}X_{it}+b_{i}+e_{it},$$

where *b_i _*is a random effect that follows $\text{N}\left(0,\sigma_{b}^{\text{2}}\right);$$\epsilon_{it}$ is an error term that follows $\text{N}\left(0,\sigma^{\text{2}}\right);$$\epsilon_{it}$ and *b_i _*are mutually independent; and ***X***_*it *_consists of the intercept, gender, smoking status, and the time-varying age. By including age into ***X***_*it*_ , we assume that the traits change linearly with time. Both visual inspection of the individual-level trait trajectories and statistical testing of the age coefficient support the linear modeling (results not shown). Because $\text{Cov}\left(Y_{it},Y_{it},\right)=\sigma_{b}^{2}+\sigma^{2}\;\text{for}\;t=t^{\prime }\;\text{and}\;\text{Cov}\left(Y_{it},Y_{it},\right)=\sigma_{b}^{2}\;\text{for}\;t\neq t^{\prime },$ the random effect *b_i _*induces a squared correlation of $\sigma_{b}^{\text{2}}/\left(\sigma_{b}^{\text{2}}+\sigma^{\text{2}}\right)$ between any pair of measurements from subject *i*. Note that *b_i _*is shared by different measurements of subject *i *so that the induced correlations are the same.

#### Families with single measurements of blood pressure

To account for the phenotype correlation among subjects from the same family, we also adopt a linear mixed-effects model. For the *i*th subject in the *p*th pedigree, we formulate that

(3) $$Y_{pi1}\text{or}Y_{pi+}=\beta S_{pi}+\gamma^{\text{T}}X_{pi}+g_{pi}+\epsilon_{pi},$$

where *g_pi _*is a random effect representing genetic similarity among family members, $\epsilon_{\text{pi}}$ is an error term that follows $\text{N}\left(0,\sigma^{\text{2}}\right),$*g_pi _*is independent of $\epsilon_{pi}$$\epsilon_{\text{pi}},$ and ***X***_*pi *_is the same as ***X***_*i *_in (1). In addition, we assume that $g_{pi}\sim \text{N}\left(0,\sigma_{g}^{2}\right),$$\text{Corr(}g_{pi},g_{pi},)=\text{2}\psi_{ii},$, where $\psi_{ii},$ is the kinship coefficient between family member *i *and *i'*. Unlike *b_i _*in (2), which is shared among correlated units (time), there is a random effect *g_pi _*for each unit (subject) here, and their covariance matrix is specified so that correlations among different pairs of family members are different.

#### Families with repeated measurements of blood pressure

Model (3) can be readily extended to accommodate repeated measurements. We include the repeated measurements in (3) as follows:

(4) $$Y_{pit}=\beta S_{pi}+\gamma^{\text{T}}X_{pit}+g_{pi}+b_{pi}+\epsilon_{pit},$$

where ***X***_*pit *_contains the time-varying age, *g*_*pi *_is introduced previously, *b*_*pi *_follows the same distribution as *b*_*i *_in model (2), and *g*_*pi *_and *b*_*pi *_are independent of each other. Unlike in (2), *b_pi _*here characterizes the additional correlation between repeated measurements after adjusting for the genetically induced portion. To see this, we consider the reduced model without *b*_*i*_:

(5) $$Y_{pit}=\beta S_{pi}+\gamma^{\text{T}}X_{pit}+g_{pi}+\epsilon_{pit}$$

For both (4) and (5), the covariance between different family members is $\text{Cov}\left(Y_{pit},Y_{pi^{\prime }t^{\prime }}\right)=\text{2}\psi_{ii},\sigma_{g}^{2}$ for any *t *and *t'*. However, the covariance between measurements $\left(t\neq t^{\prime }\right)$ from the same subject is $\text{Cov}\left(Y_{pit},Y_{pit},\right)=\sigma_{g}^{2}+\sigma_{b}^{2}$ based on (4) and $\text{Cov}\left(Y_{pit},Y_{pit},\right)=\sigma_{g}^{2}$ based on (5).

Although model (4) is more flexible than (5), the chromosome-wide scan based on (4) is not feasible within the given timeframe and available computational resources. We thus adopt a two-stage strategy that first scans chromosome 3 using (5) and then refines the *p*-values of top SNPs using (4).

#### Population stratification

GAW18 data consist of Mexican Americans from San Antonio, a population that may have an admixed ancestry of whites and Native Americans. To account for possible population stratification, we include top principal components (PCs) of SNP genotypes as covariates in the above regression models. We first obtain independent SNPs (linkage disequilibrium R^2 ^<0.2) restricted to those with MAFs 5% or greater using the unrelated subjects. Then we project the SNP loadings of unrelated subjects to their relatives to calculate the eigenvectors of the entire sample of families.

## Results

**Figure 1:**
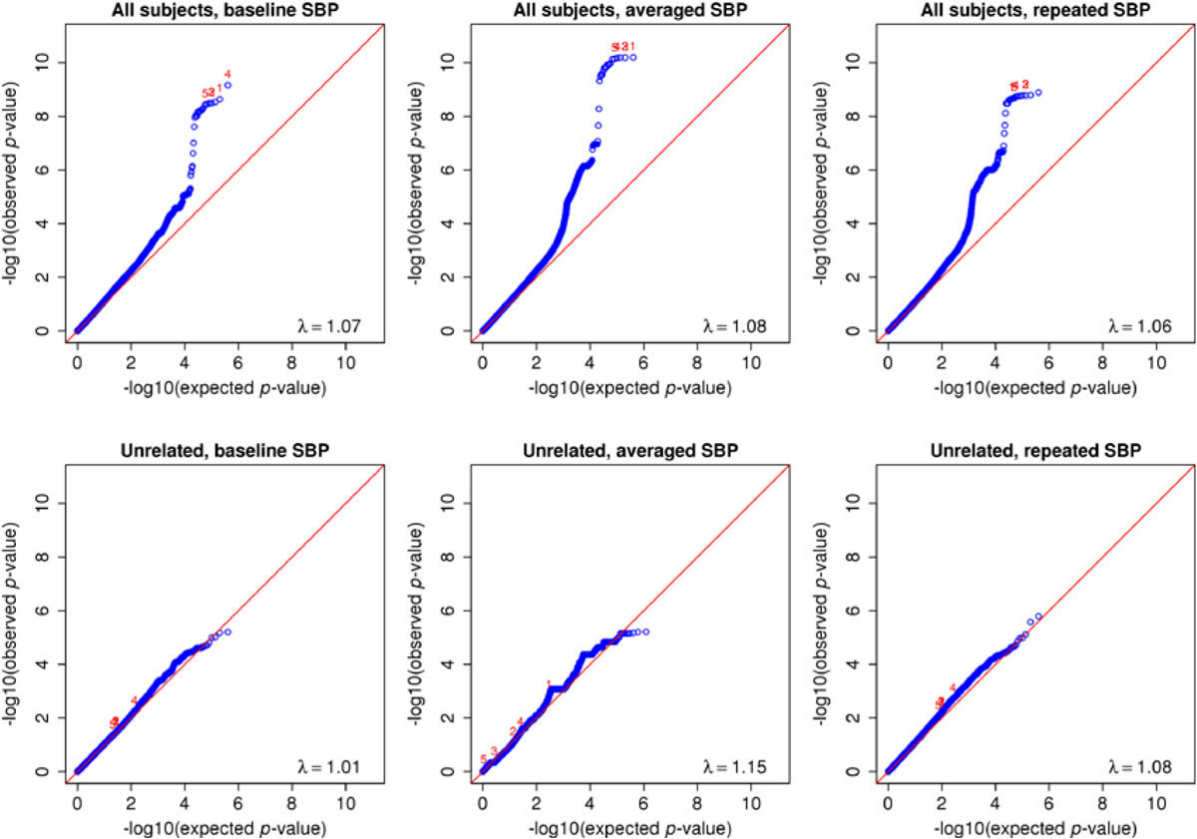
Quantile-quantile plots of *p*-values for tests between common single-nucleotide polymorphisms and systolic blood pressure (SBP)

**Figure 2:**
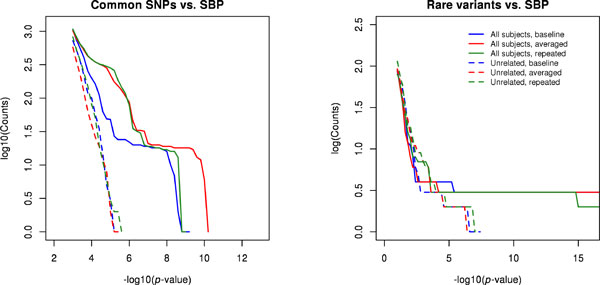
Count of tests with *p*-values lower than the *p*-value threshold across a range of *p*-value cutoffs

**Figure 3:**
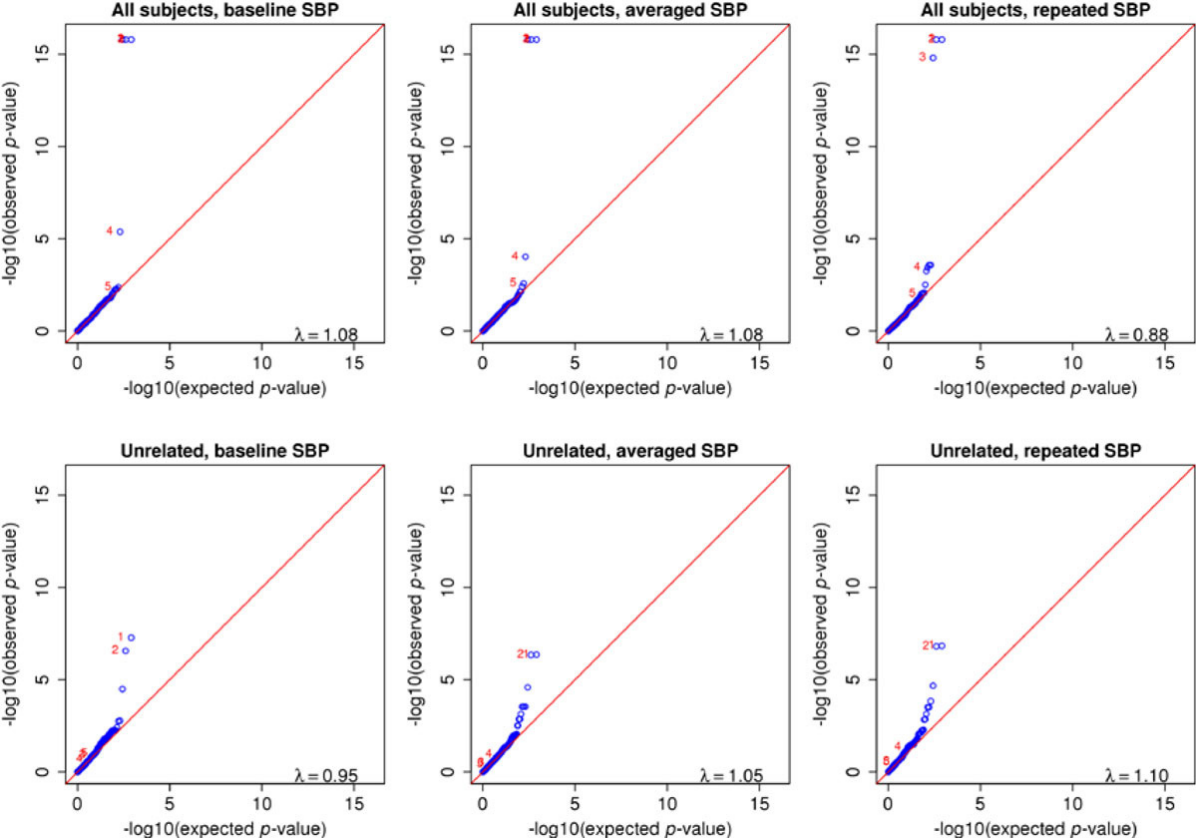
Quantile-quantile plots of *p*-values for tests between common single-nucleotide polymorphisms and systolic blood pressure (SBP)

Figure 1 displays the quantile-quantile (QQ) plots of *p*-values for testing the association between common SNPs and SBP. All 6 tests produced proper type I error because their genomic control parameter $\lambda^{\prime }$s are close to 1. This suggests that the data are well described by our models and population stratification is appropriately adjusted by the PCs. Clearly, using all pedigree-based samples is substantially more powerful than using the unrelated subjects only. In addition, using the averaged SBP yielded smaller *p*-values for top SNPs than using the baseline or repeated measurements, and using the repeated measurements is slightly more powerful than using the baseline. This pattern can also be seen in Figure 2. The top five SNPs based on the method using the averaged SBP and all subjects are listed in Table 1, whose last column provides the refined *p*-values from model (4). Note that the use of model (4) does not alter the aforementioned order based on power, although it tends to slightly improve on the use of model (5). Using the Bonferroni correction, the genome-wide significance threshold is 1.3 × 10^-7^, at which the top five SNPs can be declared as genome-wide significant by any method. Note that we only focused on chromosome 3, so what we are assessing is in fact chromosome-wide significance. For testing the association between rare variants and SBP, the 6 tests also have controlled type I error (see Figure 3 for QQ plots of *p*-values). Again, compared with the unrelated subset, the relatives added considerable information on the associations of the top three transcripts. All three types of SBP generated comparable power with all individuals, and the three consensus top transcripts are described in Table 2. Using the Bonferroni correction, the genome-wide significance threshold is 6.2 × 10^-5^, at which the three top transcripts can be declared as genome-wide significant by any method. All of the identified common and rare variants map to the gene *MAP4*, which spans from 47,892,180 to 48,130,769 on chromosome 3.

**Table 1:** p-Values for the top five common single-nucleotide polymorphisms based on the analysis of averaged systolic blood pressure

					SBP *p*-values			
					
SNP ID	Gene	Chr	Position	MAF	Baseline	Averaged	Repeated-1	Repeated-2
3_47903424	*MAP4*	3	47903424	0.124	2.3 × 10^-9^	6.3 × 10^-11^	2.2 × 10^-9^	1.1 × 10^-10^
3_47903305	*MAP4*	3	47903305	0.123	3.2 × 10^-9^	6.5 × 10^-11^	1.7 × 10^-9^	1.0 × 10^-10^
3_47905079	*MAP4*	3	47905079	0.124	3.2 × 10^-9^	6.5 × 10^-11^	1.7 × 10^-9^	1.0 × 10^-10^
3_47588649	*MAP4*	3	47588649	0.122	3.5 × 10^-9^	6.7 × 10^-11^	1.8 × 10^-9^	1.1 × 10^-10^
3_47990500	*MAP4*	3	47990500	0.123	3.6 × 10^-9^	7.2 × 10^-11^	2.1 × 10^-9^	1.1 × 10^-10^

					**DBP *p*-values**			
					
**SNP ID**	**Gene**	**Chr**	**Position**	**MAF**	**Baseline**	**Averaged**	**Repeated-1**	**Repeated-2**

3_48064367	*MAP4*	3	48064367	0.128	1.4 × 10^-11^	3.6 × 10^-13^	3.5 × 10^-13^	3.6 × 10^-13^
3_47711490	*MAP4*	3	47711490	0.120	1.8 × 10^-11^	3.9 × 10^-13^	5.4 × 10^-12^	3.9 × 10^-13^
3_48092335	*MAP4*	3	48092335	0.127	2.8 × 10^-11^	4.8 × 10^-13^	2.2 × 10^-12^	5.2 × 10^-13^
3_48105528	*MAP4*	3	48105528	0.127	2.8 × 10^-11^	4.8 × 10^-13^	2.2 × 10^-12^	5.2 × 10^-13^
3_47990500	*MAP4*	3	47990500	0.123	4.9 × 10^-11^	5.0 × 10^-13^	5.8 × 10^-12^	5.4 × 10^-13^

Note: Repeated-1 and repeated-2 correspond to model (5) and model (4), respectively.

*DBP*, diastolic blood pressure; *MAF*, minor allele frequency; *SBP*, systolic blood pressure

**Table 2:** p-Values for the top three transcripts based on the analysis of averaged systolic blood pressure (diastolic blood pressure

				SBP *p*-values			
				
Accession ID	Gene	Chr	SMAF	Baseline	Averaged	Repeated-1	Repeated-2
NM_001134364.1	*MAP4*	3	0.080	<2.2 × 10^-16^	<2.2 × 10^-16^	<2.2 × 10^-16^	<2.2 × 10^-16^
NM_002375.4	*MAP4*	3	0.074	<2.2 × 10^-16^	<2.2 × 10^-16^	<2.2 × 10^-16^	<2.2 × 10^-16^
NM_030885.3	*MAP4*	3	0.036	<2.2 × 10^-16^	<2.2 × 10^-16^	1.6 × 10^-15^	<2.2 × 10^-16^

				**DBP *p*-values**			
				
**Accession ID**	**Gene**	**Chr**	**SMAF**	**Baseline**	**Averaged**	**Repeated-1**	**Repeated-2**

NM_001134364.1	*MAP4*	3	0.080	<2.2 × 10^-16^	<2.2 × 10^-16^	<2.2 × 10^-16^	<2.2 × 10^-16^
NM_002375.4	*MAP4*	3	0.074	4.4 × 10^-16^	<2.2 × 10^-16^	<2.2 × 10^-16^	<2.2 × 10^-16^
NM_030885.3	*MAP4*	3	0.036	6.0 × 10^-15^	2.2 × 10^-16^	5.3 × 10^-15^	<2.2 × 10^-16^

Note: SMAF is the sum of minor allele frequencies (MAFs) of single-nucleotide polymorphisms included in the burden score of the gene.

DBP, diastolic blood pressure; SBP, systolic blood pressure.

The results of testing the genetic association with DBP show similar patterns as with SBP (data not shown). In particular, using the averaged DBP yielded better power than using the repeated measurements. Tables 1 and 2 provide the top common SNPs and transcripts, respectively.

## Discussion and conclusions

We investigated three approaches to exploiting longitudinal phenotype data and assessed the power gain of adding family members in the context of WGS studies. Most GWAS have focused on the population-based design, which maximizes the power per genotyped subject. Our results demonstrated that including family members can also significantly boost the power. Most GWAS have ignored the longitudinal nature of the phenotype data, which are available from many prospective cohorts. The use of the longitudinal data can provide a more accurate measurement of the phenotype and thus serves as a powerful tool in genetic association studies. With more clinic data being available through the electronic medical record (EMR) system and more clinic populations with genotypic data, the search for disease-associated common and rare variants can be more fruitful by improving the phenotyping via longitudinal information.

It appears somewhat counterintuitive that using the time-averaged measurement is more powerful than using the repeated measurement in the analysis of the GAW18 data. This is possible because, in the presence of linear time effect, the averaged measurement does not lose any information compared with the repeated measurements but simply reduces error. When more complex longitudinal structures exist, the repeated measurements retain full information and are expected to outperform the averaged measurement.

A family-based design can allow us to test association and linkage simultaneously. In this paper, we focused on the association analysis only. We modeled the association in the fixed-effect parameters and accounted for family relatedness using the random-effect parameters, whose covariances among family members are formulated through the kinship coefficient. Our models can be readily extended to linkage analysis by including another set of random-effect parameters whose covariances depend on the proportion of alleles shared identical by descent at the marker locus between a relative pair [12].

## Competing interests

The authors declare that they have no competing interests.

## Authors' contributions

YJH and YVS designed the overall study and drafted the manuscript. QH conducted statistical analyses. All authors read and approved the final manuscript.
